# Microglia in the developing retina

**DOI:** 10.1186/s13064-019-0137-x

**Published:** 2019-12-30

**Authors:** Fenge Li, Danye Jiang, Melanie A. Samuel

**Affiliations:** Department of Neuroscience, Huffington Center on Aging, Baylor College of Medicine, Houston, TX 77030 USA

**Keywords:** Microglia, Development, Retina, Synapse, Brain, Depletion models

## Abstract

Microglia are increasingly shown to be key players in neuron development and synapse connectivity. However, the underlying mechanisms by which microglia regulate neuron function remain poorly understood in part because such analysis is challenging in the brain where neurons and synapses are intermingled and connectivity is only beginning to be mapped. Here, we discuss the features and function of microglia in the ordered mammalian retina where the laminar organization of neurons and synapses facilitates such molecular studies. We discuss microglia origins and consider the evidence for molecularly distinct microglia subpopulations and their potential for differential roles with a particular focus on the early stages of retina development. We then review the models and methods used for the study of these cells and discuss emerging data that link retina microglia to the genesis and survival of particular retina cell subtypes. We also highlight potential roles for microglia in shaping the development and organization of the vasculature and discuss cellular and molecular mechanisms involved in this process. Such insights may help resolve the mechanisms by which retinal microglia impact visual function and help guide studies of related features in brain development and disease.

## Highlights

- Microglia maturation is highly specified in the retina.
- Microglia play potential roles in vascularization, neuron birth and survival, and synapse refinement.
- Diverse microglia subpopulations found in retina display distinct features.

## Background

Microglia are the resident immune cells of the central nervous system (CNS), and emerging work implicates these cells in shaping diverse features of neural development, connectivity, and homeostasis (reviewed in [1–4]). However, whether and how particular neuron or synapse types are targeted by microglia and the functional consequences of these interactions are less well described. It has been difficult to answer these questions because circuits in the brain are complex and we know relatively little about them. In this review, we discuss known microglia interactions with neurons in the accessible and well-mapped neural circuits of the mammalian retina. In the first part of the review, we present an in-depth description of the features of retina microglia and discuss their origins, localization, and organization during development. We also review evidence for microglia subpopulations and present an atlas of microglial biomarkers over development. In the second part, we discuss the functions of microglia, with a focus on their roles in modulating neurogenesis and development, particularly regarding retinal ganglion cells and astrocytes. In turn, these processes may influence novel roles for microglia in modulating neurovascular organization. Finally, we provide perspectives on key goals for future research, which include potential roles for microglia subpopulations and elucidation of mechanisms by which particular synapses are spared or removed. Continued study of microglia-specific functions in the retina may help inform related studies in the brain and provide unique opportunities to develop microglia targeted treatment strategies in diverse neurological diseases.

## Main text

### Part 1: features of retinal microglia

#### Microglia origin in the retina

Microglia originate from primitive yolk sac progenitors [5, 6]. Their development and survival are regulated by several known transcription factors and cytokine receptors (Table 1). Among these, the transcription factor PU.1 (also known as spleen focus forming proviral integration oncogene, SPI1) plays an important role in microglia development in part through its binding partner interferon regulatory factor 8 (IRF8) [7, 13–16]. Pu.1-deficient mice lack microglia, circulating monocytes, and tissue macrophages due to a reduction in early myeloid progenitors, while IRF8-deficent mice display defects in myeloid cell maturation [13, 14]. Microglia genesis is also regulated by the macrophage colony-stimulating factor receptor CSF1R. CSF1R expression on microglia is maintained throughout development. Consistent with the requirement for CSF1R expression, *Csf1r* knockout mice lack microglia in addition to yolk sac macrophages and osteoclasts [8–10]. Finally, animals lacking toll-like receptor 4 (TLR4) display reduced bipolar cell numbers and altered bipolar cell dendritic density, in addition to loss of microglia in the retina. These changes correlate with a significant reduction in retinal function, suggesting a key role for TLR4 in mediating visual function. However, whether microglia are causal to these alterations remains unclear [12].

**Table 1:** Known factors that regulate microglia formation or survival

Factors	Findings	References
PU.1	Mice were devoid of microglia in the absence of PU.1 due to a reduction in early myeloid progenitors.	McKercher et al. 1996 [7]
CSF1R	Csf1r knockout mice showed no microglia formation.	Dai et al. 2002 [8] Ginhoux et al. 2010 [9] Bruttger et al. 2015 [10]
TLR4	TLR4-deficient mice display reduced numbers of microglia in the retina.	Dando et al. 2016 [11] Noailles et al. 2019 [12]
IRF8	IRF8-deficient mice display reduced numbers of microglia during both development and adulthood.	Holtschke et al. 1996 [13] Kierdorf et al. 2013 [14]

**Fig. 1:**
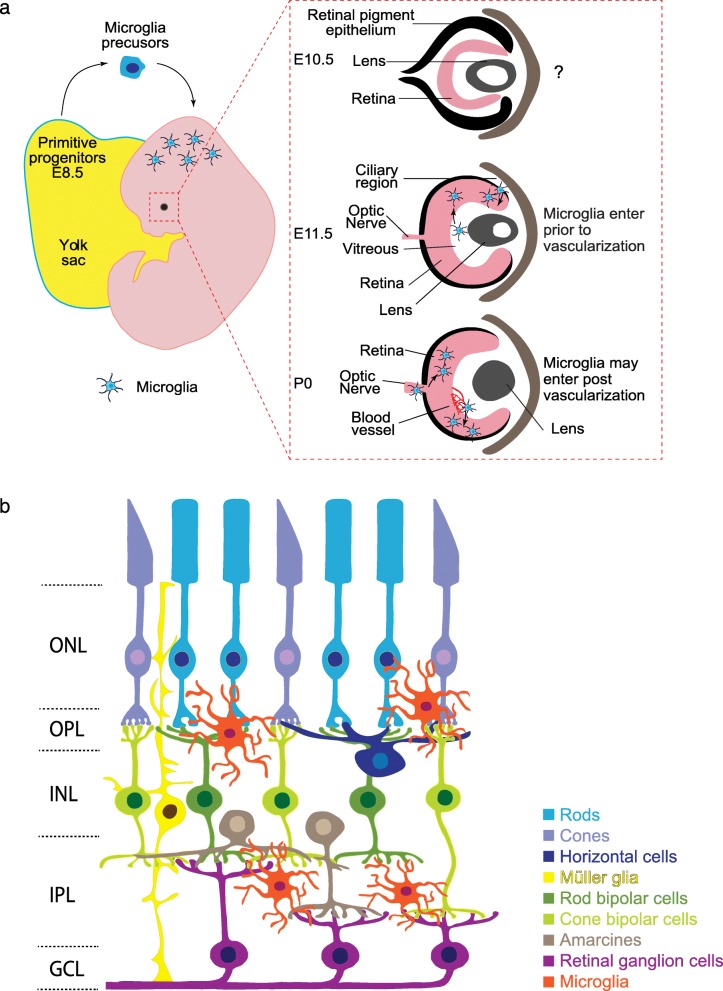
Schematic of microglia development in mouse retina. **a**. Timeline of microglia entry to the retina. Microglia are derived from primitive yolk sac progenitors and are thought to enter the CNS via the circulatory system. Microglia have been documented in the developing murine retina at E11.5 but may be present earlier. Two waves of retinal microglia infiltration have been proposed. The first wave occurs embryonically and may involve microglia entry through the vitreal retina surface or migration from the ciliary region. A second wave may involve microglia infiltration from the optic disc or via blood vessels. **b**. Schematic of the adult retina. Rod (cyan) and cone (light purple) photoreceptors reside in the outer nuclear layer (ONL) and form connections with interneurons in the outer plexiform layer (OPL). Light induced signals are then relayed to neurons in the inner nuclear layer (INL), which is comprised of horizontal cells (dark blue), Müller glia (yellow), cone and rod bipolar cells (light and dark green), and amacrines (brown). Retinal ganglion cells (magenta) receive this information through synapses in the inner plexiform layer (IPL). Their somas reside in the ganglion cell layer (GCL) along with displaced amacrine cells (not pictured). Microglia cell are found predominately in the inner retina and are largely restricted to the synaptic layers

After they differentiate, microglia home to the CNS. Microglia can be identified in mouse brain rudiment as early as embryonic day (E)8.5 ~ E9.5. They are thought to migrate to the CNS via the embryonic circulatory system as mice that lack the sodium calcium exchanger 1 (Ncx-1) have defective blood circulation and microglia fail to enter the brain [9]. The origins of microglia in the retina and their precise developmental arrival have been less well studied. They are present in human retina by 10 weeks gestation and in mouse retina by E11.5, though it is likely they arrive even earlier [17, 18]. Similar timing has been documented in other species (E7 in quail, [19]; and at E12 in rat, [20]). Two waves of retinal microglia infiltration have been proposed based on the spatiotemporal localization of these cells. The first wave happens early in development prior to vascularization (Fig. 1a). At this time, microglia are thought to enter the retina by either: 1) crossing the vitreal retina surface; or 2) migrating from non-neural ciliary regions in the periphery [17, 18, 21, 22]. A second wave of infiltration has been proposed after blood vessels have formed through invasion from the optic disc or via blood vessels themselves [23]. Since much of this evidence is correlative, firm documentation of the timing and routes by which microglia enter the retina awaits more contemporary lineage tracing approaches.

#### Microglia location and lamination in the retina

**Fig. 2:**
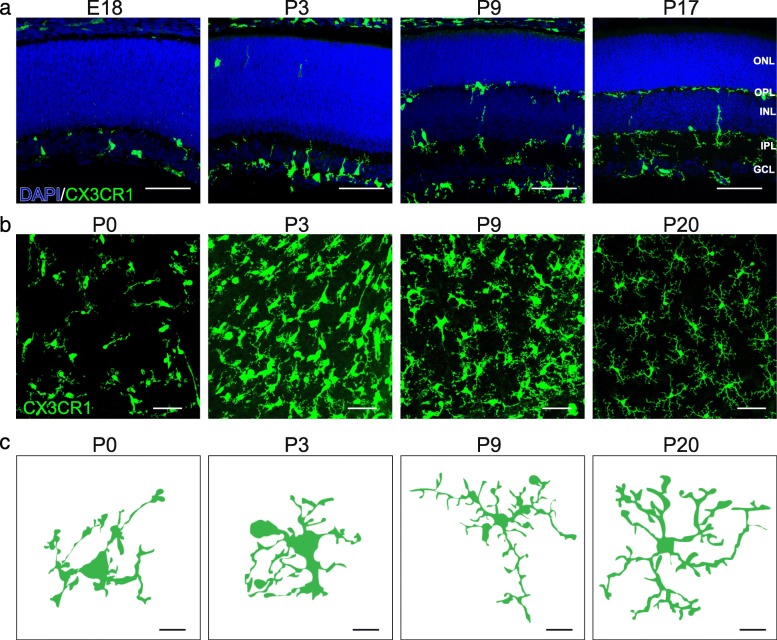
Spatiotemporal distribution of microglia in the developing mouse retina. **a**. Representative images showing distinct spatiotemporal localization patterns of microglia across retina development (E18, P3, P9, and P17) in CX3CR1^GFP/+^ mice. Microglia are highly enriched at E18 and P3 in the nascent IPL where synapses are developing. At P9, microglia also become present within the developing OPL. This pattern persists into adulthood. Blue, DAPI; green, microglia. Scale bar = 50 μm. **b-c**. Representative images (**b**, scale bar = 50 μm) and single cell reconstructions (**c**, scale bar = 10 μm) of microglia in whole mount preparations of CX3CR1^GFP/+^ retina across development (P0, P3, P9, and P20). At birth, retinal microglia are amoeboid but become progressively ramified as the retina matures

Microglia entry into the retina coincides with retinal neuron differentiation. Retinal neurons are derived from a precursor pool of retinal progenitor cells (RPCs) that divide to give rise to the five main types of retinal neurons: photoreceptors, bipolars, amacrines, horizontal cells, and retinal ganglion cells. As these neurons mature they become ordered into three cellular and two synaptic layers. Photoreceptors comprise the outer nuclear layer (ONL) and relay information through synapses in the outer plexiform layer (OPL) to inner retina neurons (horizontal, bipolar, and amacrine cells). Bipolar and amacrine cells synapse with retinal ganglion cells in the inner plexiform layer (IPL) (Fig. 1b). Microglia comprise 0.2% of total retinal cells and are found in addition to two other retina glia types, astrocytes and Müller glia [24–26]. Interestingly, microglia are predominately located in the retinal synapse layers (Fig. 2a). The adult OPL contains ~ 47% of the microglial population, while 53% are found in the IPL (Li and Samuel, unpublished). It is perhaps telling that microglia localization tracks the spatial distribution of developing retina synapses. Synapses begin to emerge as early as E17 in the nascent IPL, and at this time 99% of microglia localize to this narrow region [27, 28]. This localization persists as synapses mature and are refined. At postnatal day (P)3, ~ 80% of microglia are localized to the developing IPL and ganglion cell layer (GCL), and at P9, microglia become present within the developing OPL. This pattern persists into adulthood, with microglia and their processes localizing predominately to the inner retina and OPL, while the ONL is largely devoid of these cells (Fig. 2a) [18, 29]. Thus, microglia are at the right time and place to regulate retina synapse refinement. In line with this idea, the absolute number of retina microglia correlates with the peak of retina synapse pruning. The numbers of retina microglia increase over the first postnatal week, reaching twice that of adult levels by P7 when outer and inner retina synapses area actively refined. Microglia numbers then steadily decrease until the fourth postnatal week when they reach steady state levels and the retina circuit is considered mature [18].

#### Microglia morphology

Morphological changes in microglia are thought to correlate in part with their functional states [30–32]. Ramified microglia are often referred to as ‘resting’ while amoeboid microglia are often referred to as ‘active’ [33]. These terms can be misleading, however, as live imaging suggests that microglia are structurally dynamic in both ramified and amoeboid morphologies, though the cellular functions they carry out may differ. Ramified microglia actively retract and extend their processes, monitor neurons, and are engaged in metabolite removal and clearance in the CNS (reviewed in [3, 34, 35]). In contrast, amoeboid microglia contain numerous lysosomes and phagosomes and are thought to be engaged in synapse, axon, or cell engulfment [36, 37]. Consistent with this idea, microglia appear amoeboid in the brain during development at the peak of cell and synapse remodeling and then shift to a ramified state in the first two postnatal weeks [38, 39]. This developmental shift in microglia morphology extends to the retina. At birth, retinal microglia are amoeboid and extend their processes towards the basal side of the retina but become progressively ramified as the retina matures (Fig. 2b, c) [18]. Shifts in microglia structure also occur in response to CNS injury or pathogen invasion, leading to the formation of reactive amoeboid microglia [40, 41]. The mechanisms through which microglia alter their structural states are not well understood. Koso et al. reported that the zinc finger transcription factor Sall1 is expressed specifically in amoeboid retina microglia and that deleting Sall1 can cause ramified microglia to adopt a more amoeboid appearance [42]. Continued efforts to understand how microglia achieve different structural states and how these states impact function may aid efforts to modulate microglia activity in development or disease.

#### Microglia markers and subpopulations

**Fig. 3:**
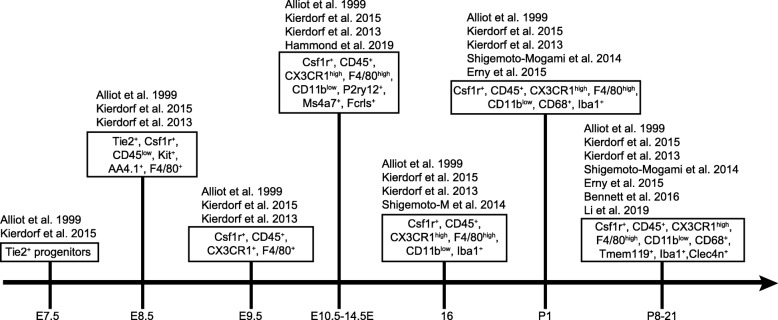
Microglia biomarkers over CNS development. A timeline is presented that summarizes biomarkers for microglia over development in the CNS. For example, Tie2 marks microglia progenitors as early as E7.5. As microglia mature they express other markers including Csf1r, CX3CR1, and Iba1. Single-cell RNA sequencing studies have also identified potential new markers such as Tmem119, Fcrls, P2ry12, and Clec4n

All microglial precursors express the common macrophage markers CX3 chemokine receptor 1 (CX3CR1) and ionized calcium binding adaptor molecule 1 (Iba1) [14, 43]. Microglia transiently express additional markers during development, including F4/80, isolectin, CD45, CD68, CD11b, and inducible nitric oxide synthase (iNOS) that are typically lost or down regulated in adult cells [6, 18, 43]. Common microglia biomarkers over development are summarized in Fig. 3 [6, 14, 44–47]. Whether microglia can be considered a group of related but distinct cell populations is an area of active investigation. One possibility is that individual microglia can display fluid cellular characteristics that vary according to developmental or disease states. Alternately, microglia may be comprised of physiologically distinct cell subsets. Progress toward resolving these questions has been somewhat challenging due to the dynamic nature of microglia, their ability to migrate, and the potential for molecular similarities between macrophages that may enter the CNS under some conditions and resident microglia populations [48, 49]. However, it is clear that antigenic, structural, and transcriptional differences exist between cohorts of microglia. For example, the cytokine IL-34 appears to demark spatially distinct populations of microglia in the retina. In normal adults, IL-34 negative microglia are mainly localized to the OPL, while IL-34 positive microglia are located in the IPL [50]. In the presence of neuron degeneration, however, both populations relocate to the retinal pigment epithelium (RPE) [50]. Retina microglia also show different levels of CD11c, CD11b, and TLR4 [11, 51, 52]. For example, CD11c appears more abundant on microglia that are localized to compromised retinal neurons [53]. Thus, it is tempting to speculate that different subsets of microglia might be tuned to perform niche specific functions or regulate specific neuron types or geographic areas of the CNS.

Several recent molecular and sequencing based profiling studies also support the presence of microglia subpopulations in brain and retina. These populations appear dynamic and vary with developmental time and the presence or absence of disease [54–56]. But some common features emerge: 1) microglia are among the most transcriptionally diverse cell types in the brain; 2) their activation states can be spatially distinct within both normal and abnormal CNS environments; and 3) developing microglia can share transcriptional similarities with those in aged or diseased environments [54–56]. In a particularly thorough study, Hammond et al. compared 76,000 individual microglia in the brain at P5 and P30 to those derived from normal, aged, and diseased adult brain [54]. This approach identified 9 transcriptional subpopulations of microglia that remained consistent across all ages and disease states. In addition, microglia derived from various regions of the developing brain showed more heterogeneity compared to those in the adult brain [54, 55]. Related studies in retina show a similar trend. Profiling of retina microglia over development revealed 6 microglia cell clusters and indicates that retinal microglia have distinct transcriptional states over development [57]. Comparison of retinal microglia to transcriptional data from brain microglia showed that a similar set of lineage specific factors are shared by both populations, suggesting that developing retina and brain microglia may be ontogenically similar. Finally, retinal microglia early in development share many common transcriptional features with retinal microglia in disease and aging, suggesting some parallels between these conditions [54]. Whether the microglia subsets in the retina and the brain represent parallel groups is presently unclear.

### Part 2: function of retinal microglia

#### Methods to study microglia function

Developing good methods to specifically alter microglia presence or function poses several challenges. First, molecules expressed on microglia are often found on macrophages or other cell types making cell-specific approaches difficult to achieve. Second, genes required for microglia development are often critically involved in other aspects of animal maturation or survival. Third, because microglia can migrate and are capable of repopulation or self-renewal, cell ablation approaches often result in at least some residual microglia and depletion drugs must be continuously administered. Due to these issues, the interpretation of microglia functional studies must take into account the models and methods used. We thus will briefly discuss the pros and cons of available microglia depletion models used to study retina and brain microglia (Table 2).

**Table 2:** Microglia depletion models

Models	Approach	Depletion age and efficiency	References
Cx3cr1^CreER^; Csf1r^F/F^	Tamoxifen administered by oral gavage at E9.5, E11.5, and E13.5	E14 (70%)	Anderson et al. 2019 [67]
Pu.1 ^-/-^ (Sfpi^−/−^)	Genetic knockout	E14 (98%)	Kierdorf et al. 2013 [14]
TGF**-**β ^−/−^	Genetic knockout	E10.5 (98%)	Butovsky et al. 2014 [58]
*Csf1r*^*-/-*^	Genetic knockout	E12.5 (98%)	Ginhoux et al. 2010 [9]
Csf1r^ΔFIRE/ΔFIRE^	Genetic knockout	E12.5 (95%)	Rojo et al. 2019 [59]
CSF1R inhibitor	PLX5622 administered to pregnant dam at E3.5	E15.5 (99%)	Rosin et al. 2018 [60]
CX3CR1^CreER^-iDTR	Tamoxifen and diphtheria toxin administered alternately (IP)	P6 (60%), P10 (95%), P14 (98%)	Puñal et al. 2019 [61]
CD11b-DTR	Inject diphtheria toxin (25ng/g dose, IP) twice at a 12h interval	P3 (10%)	Ueno et al. 2013 [62]

One category of microglia manipulation models involves deleting various effector molecules, such as complement, which are thought to alter microglia function [63, 64]. These types of models can be useful because they have more limited developmental side effects and are supported by correlative evidence implicating microglia in the phenotypes observed. Yet, in many cases, global knockouts are used that are not specific to microglia and affect other cells and systems. Such approaches do not prove the necessity and sufficiency of microglia in the observed phenotypes. To overcome this, some groups generate microglia effector molecule knockouts by crossing a *Cx3cr1*-Cre line [65] to conditional lines [65–68] though it should be noted that other cell populations are also targeted in this approach [65].

Available models to deplete or delete microglia also have important caveats. Three common approaches are used to prevent microglia formation. Each of these involves deleting or modifying one of three genes required for lymphoid or myeloid cell lineage cell development: *PU.1*, *transforming growth factor beta (TGF-β)*, or *CSF1R*. These approaches can achieve 98% microglia depletion in embryonic brain [9, 14, 58]. However, knocking out any one of these genes induces a host of additional physiologic changes that cloud the interpretation of results. Pu.1^−/−^ null mice are born alive but die of severe septicemia within days*.* Pu.1^−/−^ mice are not only devoid of parenchymal microglia in the brain, but also of circulating monocytes and tissue macrophages [14]. TGF-β1^−/−^ mice develop a lethal autoinflammatory syndrome shortly after birth and die by 3–4 weeks of age [69]. *Csf1r* null mice (*Csf1r* ^*−/−*^), Csf1 homozygous mutant mice (*Csf1* ^*op/op*^) and Csf1r specific osteoclast knockouts [TNF Receptor Superfamily Member 11a (Tnfrsf11a^cre^):Csf1r ^fl/fl^] show a lack of tooth eruption, have low body weight and growth rates, misshapen skulls, and bone defects and usually die within 30 days after birth [8, 69–71]. A new model of Csf1r modulation in which a *Csf1r* enhancer is deleted (*Csf1r*^ΔFIRE/ΔFIRE^) appears to circumvent many of these issues. *Csf1r*^ΔFIRE/ΔFIRE^ mice lack macrophages and brain microglia and are healthy and fertile up to 9 months of age without the growth and developmental abnormalities reported in *Csf1r*^−/−^ or *Csf1* ^op/op^ rodents [59].

Given these issues, many researchers have utilized microglia depletion models. Two pharmacological approaches are commonly used. Drugs that inhibit CSF1R (including PLX3397, PLX5562, GW2580, and BLZ945) can be administered in chow, in water, or intraperitoneally to deplete microglia. In the brain, this can result in 90% microglia depletion in adults, and 99% depletion at E15 when pregnant mice are fed inhibitor containing chow [60, 72]. Alternatively, liposomes containing chlodronate can be administered in vivo or in vitro to kill microglia that engulf them. While useful, this method likely targets other phagocytes as well, and the efficiency of microglia depletion is quite low (40~70%) [73, 74]. Genetic models of microglia depletion are also widely used. In these systems, depletion is achieved through targeted expression of the diphtheria toxin receptor (iDTR) primarily through crossing iDTR animals to CX3CR1-CreER animals to generate CX3CR1-CreER-iDTR mice [75]. CX3CR1 is found on microglia, as well as all monocytes, intestinal macrophages and dendric cells, some NK cells, and activated T cells [76–78]. Thus, injecting this line with alternating doses of tamoxifen and diphtheria toxin can deplete microglia but also affects subsets of other immune cells [43]. When injections are initiated at P0, this model achieves 70% microglia depletion in the retina by P6 and 98% depletion by P10–14 [61]. In brain, 99 and 85% microglia depletion are achieved after drug administration in young (~ 30 days) and adult animals (6–8 weeks), respectively [10, 79]. Following the same principle, the 10% of microglia that are CD11b positive [80, 81] can be depleted using a CD11b-DTR model [62]. Though this approach also targets other immune cell populations [82, 83]. While useful, it is important to note that these models do not achieve complete ablation, and remaining numbers of microglia can vary from animal to animal. Since it is formally possible that a small fraction of microglia could accomplish the same task as many, it is difficult to interpret negative data. Many of these models also do not allow the study of early postnatal ages since high levels of microglia depletion are not achieved for several days. Finally, these models require continual drug administration to maintain low levels of microglia since these cells can repopulate locally or from the periphery [10, 84–86].

Finally, we note that removing microglia or altering their abilities may cause both remaining microglia and other cell types to adopt different phenotypes or functions [60, 87, 88]. For example, when microglia are depleted astrocytes appear to take on the ability to modulate their own numbers during development through self-engulfment [61]. Depletion also alters remaining microglia, enabling them to rapidly repopulate. This leads to replenishment of microglia numbers within 3–7 days after acute depletion in a range of depletion systems [10, 84–86]. Where do these new microglia come from and are they comprised of the same cells as the native population? Three possibilities have been proposed: 1) they differentiate from latent microglia progenitors; 2) they are derived from residual microglia; or 3) they proliferate from peripherally invading macrophages. Evidence exists for each of these alternatives. In brain, the majority of repopulating microglia are nestin-positive (a neuroectodermal neural stem cell marker), and fate mapping analysis documented a nestin-positive microglia population that appears involved in microglia repopulation [89]. In another study, the etiology of repopulating brain microglia was investigated by comparing a Nestin-CreER:Ai14 line and a CX3CR1-CreER:Ai14 line following depletion [86]. The repopulated microglia were positive for the CX3CR1 label but negative for the nestin label, suggesting that the new cells are derived from surviving microglia (< 1%) and that these cells transiently express nestin during proliferation. Similarly, in adult retina, residual endogenous CX3CR1+ microglia near the optic nerve head were shown to undergo rapid proliferation and colonize the retina using both the CX3CR1-CreER:tdTomato and CX3CR1-CreER:Ai14 lines [90, 91]. Still, other studies have found evidence for cells that bear hallmarks of peripherally invading macrophages. Repopulating cells in which CD11b^+^ microglia had been eliminated expressed high levels of the peripheral macrophage markers CD45 and CCR2 and appeared associated with blood vessels [92]. Further, evidence suggests there could be two sources of repopulating retina microglia. In a CX3CR1-depletion model, microglia that repopulated the central retina appeard to be derived from residual microglia in the optic nerve, while microglia that repopulated the peripheral retina were suggested to arise from macrophages in the ciliary body or iris [91]. Whether repopulating microglia are transcriptionally, molecularly, or functionally similar to the native population remains an open question.

#### Microglia and retinal vascularization

In mouse, as in human, there are two phases of vascular growth in the eye. In the first phase, hyaloid vessels extend from the optic disk to the lens and supply blood and nutrients to the developing eye [93, 94]. Later in development, hyaloid vessels regress, and the retina develops its own independent vascular network [93]. Within the retina, three intraretinal vascular layers interdigitate distinct neural regions. The superficial plexus interleaves the GCL, the intermediate plexus ascends into the IPL, and the deep plexus is located within the OPL [93]. Each of these vessel layers has a characteristic location and branching pattern and thus are considered somewhat independent neurovascular units [95, 96].

Microglia have been implicated in both hyaloid vessel regression and intraretinal vascular formation in the eye via different mechanisms. Genetic or pharmacological ablation of vitreal macrophages or microglia have been shown to preserve the otherwise transient hyaloid vasculature. This process is thought to involve microglia-mediated apoptosis of vascular endothelial cells via WNT signaling [97]. After hyaloid vessels regress, endothelial cells proliferate and migrate radially into the retina from the center to the periphery, and microglia are thought to play supportive and guidance roles during this process [98]. Retinal microglia are closely apposed to endothelial tip cell filopodia, which guide blood vessel growth through the tissue [99–102]. In supporting studies, either genetic ablation or depletion of microglia reduces intraretinal vessel branching and density, while patterning was restored by intravitreal injection of exogeneous microglia [99, 103, 104]. In addition, microglia have recently been shown to regulate developmental death of astrocytes [61]. Since astrocytes form a reticular network that provides a substrate for angiogenesis and vessel patterning [105–107], microglia may also indirectly mediate vascular integrity through regulating astrocyte numbers [61]. However, it should be noted that the effects on blood vessel patterning in these microglia models are variable. In addition, there appear to be redundant mechanisms that compensate when microglia are not present which result in relatively normal adult blood vessel patterning in microglia deletion models [61, 104]. Finally, microglia have also been implicated in pathogenic retina angiogenesis. In diabetic retinopathy, abnormal intravitreal neovascularization coincides with an elevation of microglial TNF-α [108, 109]. Similar results were reported in an ischemic retinopathy model where activated retinal microglia were found to produce IL-1β, which maintained microglia activation and was associated with microvascular injury [110]. Given these observations, it is clear that much remains to be learned about the relationship between microglia and vasculature in the eye, particularly as microglia appear to alternatively promote developmental vascular regression, formation, or pathogenesis.

#### Microglia in neurogenesis and developmental cell death

Microglia have been implicated in developmental and adult neurogenesis, though the evidence remains somewhat controversial. In the retina, neurons are generated from RPCs at distinct ratios and times [111–113]. In zebrafish, targeted knockdown of Csf1r with RNAi delayed migration of microglia from the yolk sac to the retina and was correlated with a withdrawal of RPCs from the cell cycle, reduced neuron production, and microphthalmia [114]. The data in mice, however, are less clear. While application of a CSF1R inhibitor (PLX3397) [115] or minocycline (thought to reduce microglia activation, [116]) reduced RPC proliferation and viability, respectively, the numbers and gross organization of adult retina neuron cell bodies appear intact in the absence of microglia [61]. Finally, in the adult brain, microglia have been suggested to both enhance and inhibit neurogenesis, and results appear to vary depending on the model, brain region, disease state, and inflammatory and cytokine milieu [117–120].

More unambiguous studies have implicated microglia in developmental cell death of distinct retinal cell subsets. The majority of retina neuron and glia types are born in excess numbers and undergo a period of cell death from P0 to P13 [121]. While programed cell death accounts for the majority of this process [122], microglia-mediated phagocytosis plays a role in some cases. Depletion of microglia via the CX3CR1-CreER-iDTR system reduces astrocyte cell death, leading to anatomical changes in astrocyte distribution [61]. Similarly, microglia depletion in *Csf1r*^*−/−*^ mice increased the developmental density of a subset of RGCs, and complement mediated engulfment was implicated in this process [57]. Though the evidence is more limited, microglia may also be involved in initiating, sensing, or responding to canonical neural apoptosis. In the developing brain, 60% of Purkinje cells that undergo apoptosis were engulfed by or in contact with microglia [123], and developmental neuronal death appeared to facilitate microglia entry and positioning into the developing zebrafish brain [124]. The list of molecular pathways that facilitate microglia-mediated phagocytosis of neurons or CNS debris is quite extensive (Table 3) and includes synaptotagmin-11 (Syt11, [125]), G protein-coupled receptor 34 (GPR34, [126]), Mer tyrosine kinase (MerTK, [127–129]) and spleen tyrosine kinase (Syk, [130]). It remains to be determined whether these pathways converge on a central microglia phagocytic process or whether their use is context dependent.

**Table 3:** Known pathways that contribute to cell and synapse engulfment

Pathways	Findings	References
C1q/C3	Mice deficient in complement protein C1q or the downstream complement protein C3 exhibit defects in CNS synapse elimination.	Stevens et al. 2007 [63]
C3/CR3	Microglia engulf presynaptic inputs during peak retinogeniculate pruning through complement receptor 3(CR3)/C3. Microglia also regulate retinal ganglion cell elimination by CR3-mediated engulfment of nonapoptotic neurons.	Schafer et al. 2012 [1] Anderson et al. 2019 [57]
Syt11	Syt11-knockdown increased cytokine secretion and nitric oxide release in primary microglia and enhanced microglial phagocytosis.	Du et al. 2017 [125]
GPR34	GPR34-deficient microglia showed reduced phagocytosis activity in both retina and acutely isolated cortical slices.	Preissler et al. 2015 [126]
MerTK	Activated microglia release Gal-3 and a neuraminidase that desialylates microglial surfaces, enabling their phagocytosis via MerTK.	Grommes et al. 2008 [127] Caberoy et al. 2012 [128] Nomura et al. 2017 [129]
Syk	Knock down of endogenous Syk decreased microglia phagocytosis of apoptotic neurons.	Scheib. et al. 2012 [130]

#### Microglia and synapse refinement

Microglia play active roles in synapse pruning, development, plasticity, and maintenance in the developing and adult brain [131–133]. Recent data suggest that the mechanisms involved in this process may be region specific. Microglia have been shown to regulate synapse refinement in the developing retinogeniculate system via the classical complement cascade proteins C1q and C3. Genetic deletion of these complement components blocks the capacity of microglia to properly remove synapses [1, 63]. However, in the developing barrel cortex, microglia appear to eliminate synapses via CX3CR1/CX3CL1 and signaling through a disintegrin and metalloproteinase domain-containing protein 10 (ADAM10). This metalloprotease cleaves CX3CL1 into a secreted form, and mice deficient in ADAM10, CX3CR1, or CX3CL1 show decreased synapse elimination and display reduced engulfment of synapse fragments by microglia [134]. Whether these pathways and processes extend to the retina is not clear. When microglia are depleted at P5, neuron and synapse organization seem to be largely unaffected at P10 [61]. However, this negative data should be interpreted with caution since: 1) a significant fraction of retina synapse formation and remodeling occurs prior to P5; 2) the models tested thus far still retained a small fraction of microglia; and 3) visualizing microglia mediated synapse pruning at single neuron resolution may show that only particular subsets of neurons are affected. In line with these ideas, adult retina depleted of microglia using the CX3CR1-CreER-iDTR model show a loss of synapses in the outer plexiform layer over time, resulting in decreased retina function as measured by scotopic electroretinography (ERG) recordings [135]. Thus, microglia may play roles in maintaining synaptic integrity and function in the adult retina. Continued efforts to understand the role of microglia mediated synapse pruning in specific retinal neuron subsets will help resolve whether microglia may target specific cell types or synapses for removal.

## Conclusions

**Fig. 4:**
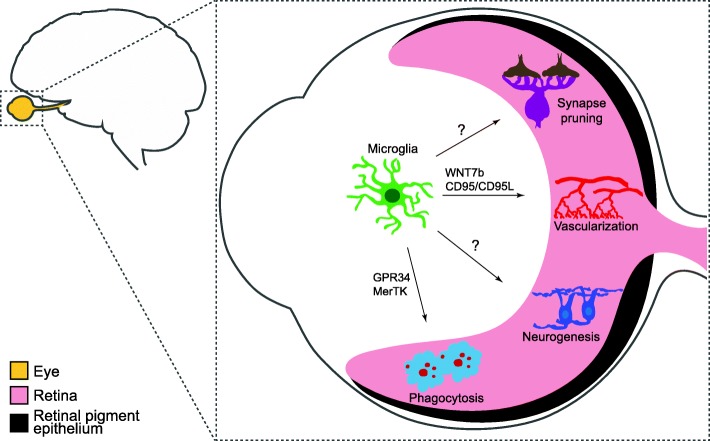
Proposed roles of microglia in the developing retina. Microglia play important roles in phagocytosis, vascularization, and neurogenesis through distinct mechanisms during retina development. These include modulation of both hyaloid vessel regression and intraretinal vascular patterning, regulation of the numbers of astrocytes and some RGC subsets, and RPC cycling. Roles for microglia in retina synapse pruning have also been proposed, though direct evidence for these pathways awaits further study

Microglia are a fascinating cell type with the potential to modulate or modify neuron development, survival, connectivity, and vascularization (Fig. 4). Studies in the retina and the brain are beginning to shed light on these processes and the mechanisms involved, but this enigmatic cell type still holds several key mysteries, including: 1) how do microglia home to the CNS and monitor and regulate their number and patterning; 2) do microglia subpopulations play region or cell-type specific roles in early neural development and neurodevelopmental disorders; 3) what are the molecular mechanisms by which microglia mediate synaptic refinement of specific neurons or synapse types; and 4) what are the interactions or signals that neurons provide to microglia that encode neuron or synapse engulfment versus sparing? Future studies that decipher these and related questions will not only enable a better fundamental understanding of neurobiology but also may provide untapped opportunities for treatment strategies aimed at preventing or reversing diverse types of neural diseases.

## Data Availability

Not applicable.

## Abbreviations

ADAM10: a disintegrin and metalloproteinase domain-containing protein 10
CNS: central nervous system
CSF1R: colony-stimulating factor one receptor
CX3CR1: CX3 chemokine receptor 1
DTR: Diphtheria toxin receptor
E: Embryonic day
ERG: Electroretinography
GCL: Ganglion cell layer
GPR34: G protein-coupled receptor 34
Iba1: Ionized calcium binding adaptor molecule 1
INL: Inner nuclear layer
IPL: Inner plexiform layer
IRF-8: Interferon regulatory factor 8
MerTK: Mer tyrosine kinase
Ncx-1: Sodium calcium exchanger 1
ONL: Outer nuclear layer
OPL: Outer plexiform layer
P: Postnatal day
PU.1: PU box binding protein
RPCs: Retinal progenitor cells
RPE: Retinal pigment epithelium
SPI1: Spleen focus forming proviral integration oncogene
Syk: Spleen tyrosine kinase
Syt11: Synaptotagmin-11
TGF-β: Transforming growth factor beta
TLR4: Toll-like receptor 4
Tnfrsf11a: TNF Receptor Superfamily Member 11a
